# Consolidating Estimates of the Incubation Period for Omicron Subvariants From the Literature and Their Comparison to the Estimate From Taiwan: A Systematic Review and Meta‐Analysis, September 2024

**DOI:** 10.1111/irv.70171

**Published:** 2025-10-30

**Authors:** Andrei R. Akhmetzhanov, Hao‐Yuan Cheng, Gillian Cheng, Jonathan Dushoff

**Affiliations:** ^1^ Global Health Program College of Public Health, National Taiwan University Taipei Taiwan; ^2^ Institute of Epidemiology and Preventive Medicine College of Public Health, National Taiwan University Taipei Taiwan; ^3^ Epidemic Intelligence Center Taiwan Centers for Diseases Control Taipei Taiwan; ^4^ Department of Biology McMaster University Hamilton Ontario Canada; ^5^ Department of Mathematics & Statistics McMaster University Hamilton Ontario Canada; ^6^ M. G. DeGroote Institute for Infectious Disease Research McMaster University Hamilton ON Canada

**Keywords:** COVID‐19, incubation period, meta‐analysis, Omicron, systematic review

## Abstract

**Background:**

The COVID‐19 pandemic was characterized by waves driven by distinct viral variants, including the Omicron variant, which emerged in October 2021. To formulate effective public health strategies and understand disease spread, accurate estimates of the incubation periods of these variants are important. Existing estimates often conflict due to biases caused by epidemic dynamics and selective inclusion of cases. Using data from Taiwan, where disease incidence remained low and contact tracing was comprehensive during the first months of the Omicron outbreak, this study aims to accurately estimate the incubation period of the Omicron (BA.1) variant incubation period.

**Methods:**

We reviewed the first 100 Omicron BA.1 symptomatic cases reported in Taiwan's contact‐tracing records (between December 2021 and January 2022). Of these, 69 had usable information. Data on exposure and symptom onset dates were fitted with the generalized gamma. A systematic search and meta‐analysis on incubation periods for Omicron BA.1/2/4/5 subvariants was then conducted to derive pooled mean estimates for the incubation periods of each subvariant.

**Results:**

The mean incubation period was estimated at 3.5 days (95% credible interval: 3.0–4.0 days), with no clear differences based on vaccination status or age. This estimate aligned closely with the pooled mean of 3.7 days (3.3–4.0 days) for Omicron BA.1 and of 3.7 days (2.3–5.1 days) for all considered Omicron variants BA.1/2 and BA.5.

**Conclusions:**

Omicron subvariants have a relatively shorter incubation period compared to previous SARS‐CoV‐2 variants. A continuous update of incubation period estimates, based on available data, is necessary to develop guidelines that can reduce the socioeconomic costs associated with COVID‐19.

## 1 Introduction

In October 2021, the emergence of the Omicron variant in South Africa marked a significant shift in the trajectory of the COVID‐19 pandemic [1, 2]. Compared to preceding variants, Omicron showed higher transmissibility but lower severity [3, 4, 5, 6] Due to increased vaccination coverage and pandemic fatigue, most nonpharmaceutical interventions were relaxed, the zero‐COVID policy was discontinued, culminating in the WHO declaring the COVID‐19 pandemic out of public health emergency on May 5, 2023 [7]. The COVID‐19 status has been downgraded in many countries, impacting their surveillance and reporting capabilities [8, 9].

Taiwan was able to contain the early spread of the Omicron variant between December 2021 and February 2022 before a larger wave began to emerge in March–April 2022 [10, 11]. COVID‐19 cases were kept below 30 daily between December 2021 and April 2022 through active case finding, contact tracing, quarantine of all close contacts, and isolation of confirmed cases. Case detection rates were likely very high during the first 3 months of the outbreak. This provides a high‐quality data set for estimates of the incubation period distribution.

Incubation periods describe the time interval between infection and the onset of symptoms and are particularly important for emerging infectious diseases as they determine the duration of quarantine or when the outbreak can be declared over. For endemic infectious diseases, they can inform the length of volunteer quarantine or help to design control measures in high‐risk settings. Given this importance, much literature has already been devoted to estimating the incubation period for various SARS‐CoV‐2 variants and Omicron subvariants (BA.1/2, BA.5). However, with downgraded classification status of COVID‐19, collecting data for accurate estimates became challenging, and much uncertainty remains.

In the earliest study, Backer et al. estimated the mean incubation period of 3.2 days (95% confidence interval [CI]: 2.9–3.6 days) for Omicron BA.1 subvariant [12]. Later studies showed similar, but slightly longer, means ranging from 3.5 to 3.8 days (95% CI: 3.2–4.1 days) [13, 14, 15]. An overall pooled mean of 3.4 days (95% CI: 2.9–4.0 days) was found by a systematic review and meta‐analysis [16]. Park et al. argued that these estimates could be biased because the data were collected during the escalating phase of the epidemic [17]. Re‐examining Backer et al. [12], they adjusted the mean to 4.2 days (95% CI: 3.6–4.9 days). Tanaka and colleagues [18, 19], on the other hand, reported comparatively shorter means of 2.9 and 3.1 days (95% CI: 2.1–4.1 days). However, their results could have been influenced by a selection bias [20, 21]. Based on contact tracing data, the authors included only cases with one‐day exposure periods, favoring shorter incubation periods. Two other studies using the same inclusion criteria reported even shorter means [22, 23].

The present study examines the dataset based on epidemiological reports from active case finding and contact tracing conducted in Taiwan in the early phase of Omicron spread (December 2021–January 2022). This period, characterized by low incidence and likely high case detection rates, allowed us to reliably identify individual exposure times and symptom onset dates for a subset of cases. The dynamic bias highlighted by Park et al. [17] was unlikely to be important because incidence changes were slow, whereas the effect of the selection bias was reduced by using broader case inclusion criteria when cases with exposure windows longer than one were also eligible for the analysis.

Our study provides an opportunity to derive potentially less biased estimates, since other studies conducted during the Omicron era were often constrained by either possible dynamic effects such as the rising number of cases or the inability to provide detailed contact tracing records. Moreover, it is essential to compare our estimates with those from the literature, for example, using a pooled mean derived from a systematic search and meta‐analysis. Meta‐analysis can be used, in this context, to verify the validity of the studies and to assess the risk of bias.

## 2 Methods

### 2.1 Data

Data used in this study were obtained through the analysis of daily public and internal epidemiological reports from the Taiwan Centers for Disease Control (CDC) and press conferences of the Central Epidemic Command Center (CECC) [24, 25]. All records were based on epidemiological investigations conducted by the Taiwan CDC and local Taiwan health authorities. All cases reported from December 27, 2021, the date of the first report of a local Omicron case in Taiwan, until January 18, 2022, were considered for inclusion in the analysis. Data records were compiled by H.‐Y.C. and cross‐verified by A.R.A.

Following active case finding and contact tracing efforts implemented in Taiwan during that period, it was assumed that high levels of case ascertainment were achieved in this study [26, 27]. Because all cases were followed for up to 14 days after their last exposure [28], there was a reduced risk of misclassifying cases as asymptomatic due to shorter follow‐up times [29].

Out of 128 cases reported during this period, one case confirmed with Delta variant and 27 cases with missing data on the date of symptom onset were excluded from our study. Among the remaining 100 cases, 94 were sequenced and genetically identified as Omicron BA.1.1.5, and 6 were epidemiologically linked to a case with a known Omicron infection. The data, being interval‐based, set constraints on exposure, $e_{i}$, and symptom onset time, $o_{i}$, such that $E_{L,i}\leq e_{i}\leq E_{R,i}$ and $O_{L,i}\leq o_{i}\leq O_{R,i}\equiv O_{L,i}+1\;\mathrm{day}$ for each case i. When the right end of the exposure interval, $E_{R,i}$, was unknown or if it was later than the right end of the symptom onset interval, $O_{R,i}$, it was assigned to that bound (i.e., $E_{R,i}≔O_{R,i}$). If the right end of the exposure interval coincided with the right end of symptom onset interval ($E_{R,i}\equiv O_{R,i}$), and the left boundary, $E_{L,i}$, was unknown, the case was excluded from the analysis for being noninformative for the estimation; there were 31 such cases. Of the remaining 69 cases finally included in the study, 65 had definitive exposure time intervals and 4 cases were left‐censored (Figure S1).

### 2.2 Statistical Framework

The data were fitted to the generalized gamma distribution (GGD). The GGD, which is defined by the following three parameters: shape, location, and scale, is known to represent three commonly used distributions: gamma (shape equals the scale), Weibull (shape equals one), and lognormal (shape equals zero) [30, 31]. However, the use of GGD comes with a cost of wider posterior credible intervals (CrI) for model parameters [30]. Its fit was also compared to each of the standalone gamma, Weibull, lognormal distributions and their mixture (Supplementary Materials A, B).

The summary statistics were then aggregated for the subgroups accounting for vaccinated and nonvaccinated cohorts as well as considering three age groups: those under the age of 18, those over 50, and those in between. The mean, interquartile range (IQR), and 95% credible intervals (95% CrI) were identified by exploring the individual posterior incubation periods for each of the subgroups.

### 2.3 Systematic Review and Meta‐Analysis

To collect data on the estimation of the incubation period for Omicron subvariants, we searched PubMed/MEDLINE and Embase web‐based databases from the presumptive inception of the Omicron variant on November 1, 2021, to August 19, 2024. The search strategy was based on a direct word search approach (Supplementary Materials C). The queries were designed to identify Omicron‐related publications from a collection consistent with COVID‐19 transmission literature and with creation dates starting from November 1, 2021, when the Omicron variant presumably emerged. We also incorporated generation time and serial interval in our search, as some studies, which focused on these intervals also reported estimates of the incubation period. The search was verified independently by two researchers (A.R.A. and H.‐Y.C.).

Following selection of relevant studies, we implemented a random‐effects model to perform the meta‐analysis comparing Omicron variants BA.1, BA.2, and BA.5 [32]. No sources on incubation periods of Omicron variants emerging later than BA.5 were found. The quality assessment was conducted by using a slightly revised version of the protocol proposed by McAloon and colleagues [33] and adopted for their review of incubation periods of the ancestral SARS‐CoV‐2 variant (Supplementary Materials D). The risk of bias was assessed by G.C.

### 2.4 Technical Details

The incubation period distribution and meta‐analysis were performed within a Bayesian framework using Markov Chain Monte Carlo (MCMC) sampling techniques. Bayesian estimation was implemented using Stan software (version 2.36.0; Stan Development Team). Each run of simulations was composed of 4 parallel chains with 15,000 posterior draws, including 2500 draws used for tuning and disregarded for the final output. The convergence of simulations was verified by ensuring the Gelman‐Rubin R‐hat statistic was below 1.01 [34] and convergence was also visually inspected. The code is available at http://github.com/aakhmetz/Omicron‐incper‐Taiwan‐2023.

## 3 Results

### 3.1 Taiwanese Cohort

Sixty‐nine case records were analyzed, with an average patient age of 35.4 years (range 1–66 years), and with females constituting 62% (43 cases) (Table 1). The vaccination status for 13 cases (18.6%) was unknown. Of the remaining 56 cases, 40 (71.4%) were breakthrough infections, with the majority of vaccinees having received two doses of the ChAdOx1 nCoV‐19 vaccine (AZD1222). The mean age of vaccinated individuals was 40.3 years (range: 17–66) and of nonvaccinated individuals was 22.6 years (range: 1–57). The younger age in the nonvaccinated group is at least partly due to vaccination guidelines.

**TABLE 1:** Demographic characteristics of the studied cohort of patients. The values indicated by counts and their relative fraction are indicated in the parenthesis.

	Total sample	Nonvaccinated	Vaccinated	Unknown
No. cases	69	16	40	13
Sex (%)				
Male	26 (37.7%)	7 (43.8%)	15 (37.5%)	4 (30.8%)
Female	43 (62.3%)	9 (56.2%)	25 (62.5%)	9 (69.2%)
Age group (%)				
0–17 years old	8 (11.6%)	7 (43.8%)	1 (2.5%)	0 (0.0%)
18–49 years old	45 (65.2%)	7 (43.8%)	27 (67.5%)	11 (84.6%)
50 + years old	16 (23.2%)	2 (12.5%)	12 (30.0%)	2 (15.4%)

**FIGURE 1:**
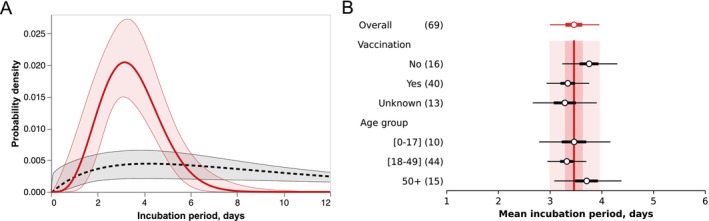
Estimation of the incubation period for Omicron BA.1 using Taiwanese cohort, December 2021–January 2022. (**A**) shows estimated incubation period distribution for the Omicron BA.1 variant compared to the earlier estimate for the pre‐Alpha (ancestral) variant [35]. Solid red line shows the posterior median for Omicron variant, dashed grey line shown the posterior median for pre‐Alpha variant. Shaded areas show the 95% credible interval (CrI) (**B**) shows the mean incubation period stratified by vaccination status or age group compared to the overall estimate, which is shown by red on top. Whiskers indicate 95% CrI, while thicker lines indicate 50% CrI. The vertical red line reiterates the overall mean, while the light shaded area shows its 95% CrI, and the dark shaded area shows its 50% CrI.

The estimated distribution of the incubation period and its comparison with the ancestral (pre‐Alpha) variant based on data from Wuhan, China, is shown in Figure 1A. The estimated mean was 3.5 days (95% CrI: 3.0–4.0 days) and the SD 1.3 days (95% CrI: 1.0–1.8 days), with the 95th percentile mean posterior predictive at 5.8 days. Among the three distributions, the lognormal was preferred over the gamma and Weibull distributions (mean relative weight 0.65). Generally, the mean and SD of all three distributions as well as GGD were fairly similar (Figure S2, Table S1). Upon stratifying the patient cohort by vaccination status or age group, no clear differences were found, although vaccinated or middle‐aged adults (18–49 years of age) tended to have shorter incubation periods (Figure 1B).

### 3.2 Systematic Review and Meta‐Analysis

**FIGURE 2:**
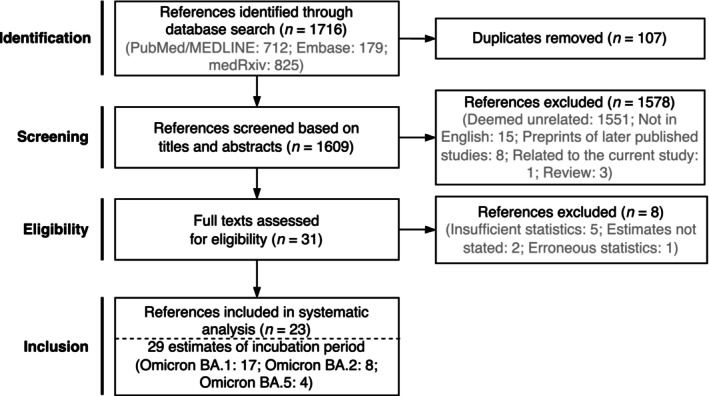
Flow diagram for identifying and selecting published studies for our meta‐analysis of incubation period for Omicron variants.

Out of 1716 references obtained from the literature search, 23 references were selected for our meta‐analysis after deduplication and manual inspection (Figure 2). This selection provided 29 estimates of the incubation period for three Omicron variants BA.1, BA.2, and BA.5. Seventeen estimates were reported for the incubation period for the Omicron BA.1 variant [12, 13, 15, 17, 18, 19, 22, 36, 37, 38, 39, 40, 41, 42, 43, 44]. Eight were provided for Omicron BA.2 [38, 39, 40, 41, 42, 43, 44, 45], and four were reported for Omicron BA.5 [19, 46, 47, 48]. These were used for comparison with our BA.1 estimates and to update earlier reviews.

**FIGURE 3:**
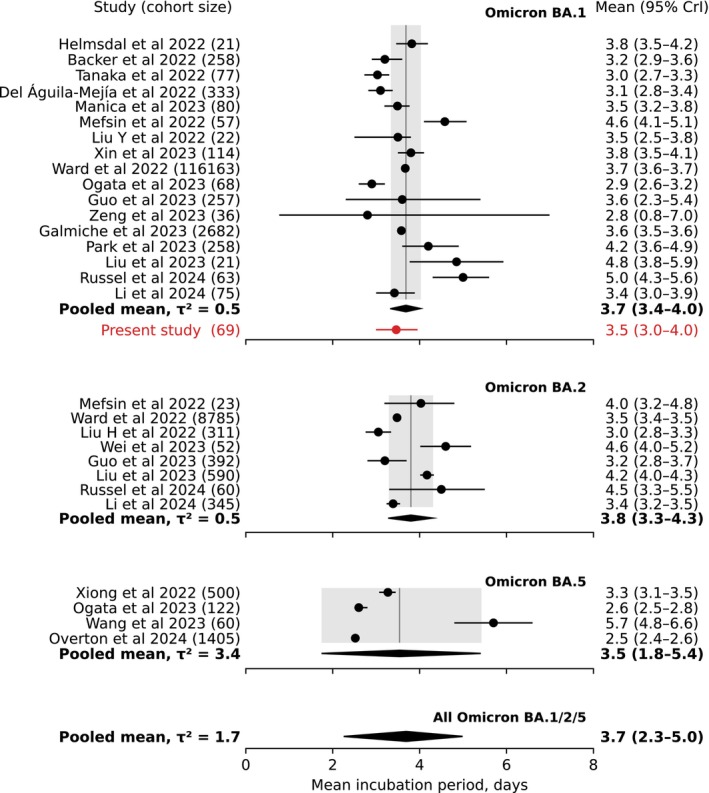
Meta‐analysis of the mean incubation periods for Omicron variants BA.1, BA.2, and BA.5. The pooled mean is indicated in bold, while the estimate of the present study is indicated in red, and it was not a part of the meta‐analysis.

**FIGURE 4:**
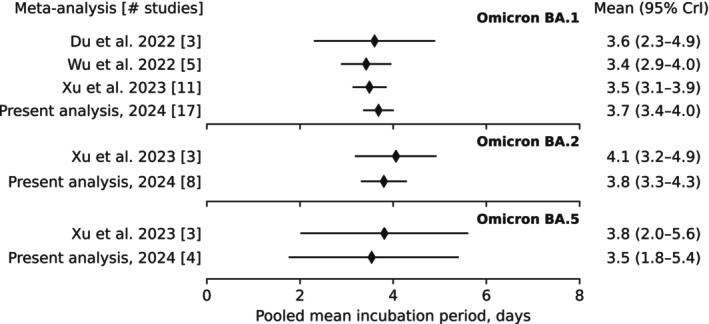
Comparing results of previously published systematic reviews and meta‐analyses with the present analysis. The pooled means across various SARS‐CoV‐2 variants were extracted from three meta‐analyses (Du et al. [49], Wu et al. [16], and Xu et al. [50]). The diamond dot indicates the posterior median, while whiskers indicate the 95% credible interval.

The meta‐analysis estimated a pooled mean for Omicron BA.1 at 3.7 days (95% CrI: 3.4–4.0 days), closely aligning with our Taiwan estimate (Figure 3). The pooled estimates for both Omicron BA.2 (3.8 days [95% CrI: 3.3–4.3 days]) and Omicron BA.5 (3.5 days [95% CrI: 1.8–5.4 days]) were close but with larger uncertainty. The overall pooled mean was estimated at 3.7 days (95% CrI: 2.3–5.0 days). This pooled mean was consistent with other previously published meta‐analyses, which included a smaller number of selected studies (Figure 4). When we restricted the analysis to studies with a quality score of four or greater (out of six), the pooled estimates changed only slightly, while the degree of heterogeneity among the studies was reduced (Figure S3).

## 4 Discussion

Using Taiwan data, we estimated the incubation period distribution for Omicron BA.1 infections, finding a mean of 3.5 (3.0–4.0) days and a SD of 1.3 (1.0–1.8) days. These estimates were consistent with our meta‐analysis based on an updated systematic review (updated on August 19, 2024), and with three earlier meta‐analyses.

Our primary data were collected in Taiwan between December 25, 2021, and January 18, 2022, a period during which the daily occurrence of COVID‐19 was less than 20 cases and the epidemic was not in an escalating phase. These high‐quality data are the result of careful case‐finding and contact‐tracing efforts of Taiwan's CDC and local health authorities. Given that the contact tracing teams were not overwhelmed during that period, and based on personal communications, we believe that ascertainment rates were high.

Our analysis did not find clear differences in incubation periods between vaccinated and nonvaccinated individuals. Notably, the majority of vaccinated cases had received the ChAdOx1 nCoV‐19 (recombinant) vaccine (AZD1222), which has been reported to demonstrate rather limited protection against symptomatic disease caused by the Omicron variant. It was therefore difficult to assess the effects of vaccination on the incubation period and severity associated with the infection. We also found no clear difference in the incubation period across different age groups.

In our data collection and estimation process, we avoided restrictive case inclusion criteria such as selecting only those with a 1‐day exposure window, in an attempt to minimize potential selection bias. Given this approach, coupled with the fact that our data collection period occurred during a stable, low‐incidence phase of the outbreak, we believe that our estimate is less prone to the biases associated with selection and dynamic changes.

We acknowledge several limitations. First, the possibility of selection biases influencing our results cannot be dismissed. Second, the demographic and social composition of our study cohort may differ from the general population. For instance, an initial outbreak hotspot was among staff at an international airport and a COVID‐19 prevention hotel. Third, our meta‐analysis did not incorporate a rigorous quality assessment of the included studies. The development of methodologies for quality assessment remains an active field of research. An initial attempt was made by McAloon et al. [33] for their rapid meta‐analysis of ancestral (pre‐Alpha) incubation periods, though one of their criteria, favoring a strict selection of cases with a one‐day exposure window, may bias estimates downward. Notably, none of the studies included in the meta‐analysis of McAloon et al. met this criterion. Previous studies investigating the 2014–2016 Ebola outbreak in West Africa have underscored that including cases with a variety of exposure windows reduces bias in estimates of the incubation period [51, 52].

The increased transmissibility of new SARS‐CoV‐2 variants, along with the discontinuation of active case finding and contact tracing efforts, has complicated the estimation of the incubation period and other epidemiological characteristics of SARS‐CoV‐2. A larger degree of heterogeneity and fewer studies dedicated to estimating the incubation period of Omicron BA.2 and BA.5 variants can be observed (see bottom panel in Figure 4). However, alternative methods that do not rely on contact tracing data have recently been utilized for estimating the incubation period. Ejima et al. previously used the viral load data to estimate the ancestral incubation period [53]. Recently, Russel et al. employed a combined analysis of viral kinetics and incubation periods for various SARS‐CoV‐2 variants [43]. Their model estimated the incubation period of Omicron BA.1 infections with a mean of 5.0 days (95% CI: 4.3–5.6 days; 63 cases), which is longer than both our estimate and the reported pooled means.

One of the strengths of our study is that while estimating the incubation period, we minimize the risk of bias caused by an exponential growth of cases. The time period for data collection was chosen during a stable phase of the epidemic at the beginning of 2022 and when the control measures such as case finding and contact tracing were still implemented. We show that our estimates still confirm the previously accumulated evidence that Omicron variants possess shorter incubation periods with probable means around 3–4 days. In our study, we also avoid a bias caused by a narrower case selection when only cases with one‐day exposure windows are included in the analysis [20]. We adopt a more flexible approach by allowing multiday exposure windows. However, our estimates are still prone to recall bias when patient's memory on symptom onset and exposure dates may fade over time [54, 55]. Despite that the mathematical modeling framework may leverage this bias, its validation and application in practice are still uncertain.

Nonetheless, we accumulated evidence that the incubation period for Omicron variants is likely to be around the mean of 3.7 days. Our estimation for the incubation period for the Taiwan cohort in December 2021–January 2022 that followed a detailed epidemiological investigation resulted in a similar estimate of 3.5 days. Whereas the mean could be identified accurately for an early emergent BA.1 variant, the assessment became more challenging for later emergent variants BA.2 and BA.5, which was indicated by a wider uncertainty interval.

In conclusion, our study provides a detailed estimate of the incubation period for the Omicron BA.1 variant of SARS‐CoV‐2 by leveraging high‐quality data from Taiwan during a period of low incidence. These results should prove useful for public health planning and interventions, particularly in the context of evolving SARS‐CoV‐2 variants. Future studies, including alternative methods that do not rely on contact tracing data, will be essential to continue refining our understanding of the incubation period and other epidemiological parameters for new and emerging SARS‐CoV‐2 variants.

## Author Contributions

**Andrei R. Akhmetzhanov:** conceptualization, methodology, software, data curation, investigation, formal analysis, supervision, funding acquisition, visualization, writing – review and editing, writing – original draft, resources, project administration, validation. **Hao‐Yuan Cheng:** conceptualization, data curation, investigation, writing – review and editing, validation, methodology, investigation, writing – review and editing. **Gillian Cheng:** conceptualization, data curation, investigation, writing – review and editing, validation, methodology, investigation, writing – review and editing. **Jonathan Dushoff:** conceptualization, methodology, investigation, writing – review and editing, supervision.

## Ethics Statement

This study was approved by the Research Ethics Committee of National Taiwan University (202304HM020).

## Conflicts of Interest

The authors declare no conflicts of interest.

## Peer Review

The peer review history for this article is available at https://www.webofscience.com/api/gateway/wos/peer‐review/10.1111/irv.70171.

## Supporting information


**Table S1:** Posterior predictive incubation period and estimated model parameters for each distribution (first column). The values are the posterior means, while the 95% credible intervals are shown in the parenthesis. GGD stands for the generalized gamma distribution.
**Table S2:** Estimated parameters of the incubation period using mixture model, stratified by vaccination status or by age group. The values are shown by their posterior medians and 95% credible intervals indicated in the parenthesis.
**Figure S1:** Data on confirmed local COVID‐19 cases associated with Omicron BA.1 variant that was collected in Taiwan from 25 December 2021 through 18 January 2022. (A) shows the exposure windows (in orange) and symptom onset days (in black) for 69 cases included in our study. The four cases below the dashed line have left‐censored exposure windows (below). (B) shows the epidemiological curve for symptomatic COVID‐19 cases by their date of symptom onset. Black bars indicate included cases, white bars show cases excluded for insufficient data, and hatched bars cases reported after the cutoff date of January 18, 2022. The horizontal axis is shared among both panels (A) and (B). Cases associated with the COVID‐19 cluster among migrant workers were omitted in (B) as non‐community related.
**Figure S2:** Comparing the estimates of incubation period across various models. The incubation period was fit to the generalized gamma distribution (GGD), standalone gamma, Weibull, or lognormal distribution, or the mixture of last three. The last column shows relative weights for each standalone distribution determined by the inference of the mixture model. Their sum was normalized to one. Inside the violin plots, the circles show the estimated posterior median, thick lines show the interquartile ranges and thin whiskers show the 95% credible intervals. Each value in the table indicates the posterior mean and 95% credible interval that are shown in parenthesis.
**Figure S3:** Meta‐analysis of the mean incubation periods for Omicron variants BA.1, BA.2, and BA.5 when only studies quality assessment scores above four were selected. The pooled mean is indicated in bold, while the estimate of the present study is indicated in red, and it was not a part of the meta‐analysis.

## Data Availability

All data used for this study can be found at: http://github.com/aakhmetz/Omicron‐incper‐Taiwan‐2025.
