# Supplementary material for: Consolidating Estimates of the Incubation Period for Omicron Subvariants From the Literature and Their Comparison to the Estimate From Taiwan: A Systematic Review and Meta‐Analysis, September 2024
Source: Influenza Other Respir Viruses. 2025 Oct 30;19(11):e70171. doi: 10.1111/irv.70171 (PMC12573278; doi:10.1111/irv.70171)
Supplement: Supplementary file 1 — Table S1: Posterior predictive incubation period and estimated model parameters for each distribution (first column). The values are the posterior means, while the 95% credible intervals are shown in the parenthesis. GGD stands for the generalized gamma distribution. Table S2: Estimated parameters of the incubation period using mixture model, stratified by vaccination status or by age group. The values are shown by their posterior medians and 95% credible intervals indicated in the parenthesis. Figure S1: Data on confirmed local COVID‐19 cases associated with Omicron BA.1 variant that was collected in Taiwan from 25 December 2021 through 18 January 2022. (A) shows the exposure windows (in orange) and symptom onset days (in black) for 69 cases included in our study. The four cases below the dashed line have left‐censored exposure windows (below). (B) shows the epidemiological curve for symptomatic COVID‐19 cases by their date of symptom onset. Black bars indicate included cases, white bars show cases excluded for insufficient data, and hatched bars cases reported after the cutoff date of January 18, 2022. The horizontal axis is shared among both panels (A) and (B). Cases associated with the COVID‐19 cluster among migrant workers were omitted in (B) as non‐community related. Figure S2: Comparing the estimates of incubation period across various models. The incubation period was fit to the generalized gamma distribution (GGD), standalone gamma, Weibull, or lognormal distribution, or the mixture of last three. The last column shows relative weights for each standalone distribution determined by the inference of the mixture model. Their sum was normalized to one. Inside the violin plots, the circles show the estimated posterior median, thick lines show the interquartile ranges and thin whiskers show the 95% credible intervals. Each value in the table indicates the posterior mean and 95% credible interval that are shown in parenthesis. Figure S3: Meta‐analysis of the mean [file IRV-19-e70171-s001.pdf]

Supplementary Materials

A. PARAMETERS OF THE GENERALIZED GAMMA DISTRIBUTION (GGD)

Three parameters are identified with defining the GGD: shape (a), location (μ), and scale (σ). The probability density function is defined by the expression:

$$f(x;\theta \coloneqq \{a,\mu,\sigma\}) = \frac{a}{x\sigma\Gamma(a^{-2})}[s(x)]^{a^{-2}} \exp\big(-s(x)\big)$$

where:

$$s(x) = a^{-2} \exp\left(\frac{a}{\sigma}(\ln x \ -\mu)\right)$$

and $\Gamma(\cdot)$ is the gamma function. Whereas the cumulative distribution function is defined as follows:

$$F(x;\theta) = \frac{\gamma\big(a^{-2}, s(x)\big)}{\Gamma(a^{-2})}$$

where $\gamma(\cdot)$ is the lower incomplete gamma function.

The mean, m_{inc}, and standard deviation (SD), s_{inc}, can be then determined by the formulas:

$$m_{inc} = \exp\left(\mu + \frac{2\sigma}{a}\ln a\right)\frac{\Gamma(a^{-2}+\sigma/a)}{\Gamma(a^{-2})}$$

$$s_{inc} = m_{inc}\left[\frac{\Gamma(a^{-2})\Gamma(a^{-2}+2\sigma/a)}{\big(\Gamma(a^{-2}+\sigma/a)\big)^2} - 1\right]^{1/2}$$

B. ESTIMATION PROCEDURE

To estimate the distribution of the incubation period, we fitted the data to GGD, one of three commonly used distributions: gamma, Weibull, and lognormal, and their mixture. In each instance, the distribution was parameterized by its mean and SD. Whereas, the GGD had an additional parameter such as a shape. The log-transformed mean and SD were imposed with weakly informative priors using a normal distribution with zero mean and SD of two. In this regard, the 95% prior interval was concluded between 0.02 and 48.1 days. The same weakly informative prior was imposed on an additional third parameter of the GGD, the log-transformed inversed square of the shape ($\ln a^{-2}$). Such choice of the third parameter ($\ln a^{-2}$ rather than $\ln a$) was directed by a better observed numerical stability of Monte-Carlo Markov chain (MCMC) simulations.

If the probability density function (PDF) is $f(x;\theta)$ and cumulative distribution function is $F(x;\theta)$, where θ is a set of distribution parameters, the likelihood function, which is doubly censored and right truncated at $T_{inc} = 18\ \text{January}\ 2022$, can be defined by the formula [56]

$$L\big(\theta; D \equiv \{E_{L,i}, E_{R,i}, O_{L,i}, O_{R,i}\}\big) = \prod_i \iint_{\Phi_i} \frac{f(o_i - e_i;\theta)}{F(T_{inc} - e_i + 1;\theta)} do_i de_i$$

Here, e_i and o_i are randomly uniformly distributed within the area in the state space

$$\Phi_i \coloneqq \big[\{o_i, e_i\}: O_{L,i} \le o_i \le O_{R,i}, E_{L,i} \le e_i \le \big(\{o_i, E_{R,i}\}\big)\big]$$

When the exposure period of a case i was left-censored, the left bound $E_{L,i}$ was imposed with a prior, with the difference $E_{R,i} - E_{L,i}$ following the exponential distribution with the mean of ten, and implying that exposure event e_i would likely occur within ten days before $E_{R,i}$.

Additionally, we fitted the data to one of three standalone distributions: gamma, Weibull, and lognormal. Similarly to GGD, each distribution was parameterized by its mean and standard deviation (SD). The respective probability density function (PDF) was denoted as $f_l(x;\theta)$ and cumulative distribution function was denoted as $F_l(x;\theta)$ ($l = 1,2,3$). The likelihood function was represented as:

$$L_l\left(\theta; D \equiv \left\{E_{L,i}, E_{R,i}, O_{L,i}, O_{R,i}\right\}\right) = \prod_i \iint_{\Phi_i} \frac{f_l(o_i - e_i;\theta)}{F_l(T_{inc} - e_i + 1;\theta)} do_i de_i,$$

To convey selection between gamma, Weibull, and lognormal distribution, we employed the Bayesian mixture model framework. In this context, the likelihood function was defined by a sum of three component likelihoods L_l, each weighted by w_l ($\sum_l w_l = 1$):

$$L(\theta; D) = \sum_{l=1,2,3} w_l L_l(\theta, D),$$

with noninformative priors imposed on weights, w_l. The posterior predictive probability for selecting the distribution l was determined by expression: $P_l = w_l L_l(\theta, D)/L(\theta, D)$.

The results of their comparison are shown in Supplementary Figure 2.

C. DATABASE SEARCH STRATEGIES

August 19, 2024

PubMed/MEDLINE <2021 November 1 to 2024 August 19>

---

1. Omicron
2. (COVID-19) OR (SARS-CoV-2)

3. (incubation) OR (generation time) OR (serial interval)
4. "2021/11/01"[Date - Create] : "2024/08/19"[Date - Create]
5. (1) AND (2) AND (3) AND (4)

Embase <2021 November 1 to 2024 August 19>

---

1. 'Omicron'
2. 'COVID-19' OR 'SARS-CoV-2'
3. 'incubation' OR 'generation time' OR 'serial interval'
4. [01-11-2021]/sd
5. (1) AND (2) AND (3) AND (4)

medRxiv <2021 November 1 to 2024 August 19>

---

1. 'Omicron'
2. 'COVID-19' OR 'SARS-CoV-2'
3. 'incubation' OR 'generation time' OR 'serial interval'
4. (1) AND (2) AND (3)

D. QUALITY ASSESSMENT FOR INCLUDED STUDIES

First, we describe the used quality assessment scale, which was based on Newcastle-Ottawa quality assessment scale for observational non-randomized studies in meta-analyses and was subsequently revised by McAloon *et al.* [33]. Comparing to the protocol of McAloon *et al.*, we slightly modified the following three points: “Ascertainment of exposure”, “Ascertainment of outcome”, and “Precision of the

exposure window for cases used in final analysis”, and added a new point “Adjustment for epidemic phase”. In two instances of “Ascertainment of exposure / of outcome”, a new option of collecting data through a web-based interview was added. Whereas, the priority of options for “Precision of the exposure window for cases used in final analysis” were re-arranged following discussion in Cheng *et al.* [20] Two remaining points “Representativeness of the study cohort” and “Precision of estimate of outcome” were unchanged.

<u>External validity</u>

A. Representativeness of the study cohort

1. **no selection of cases based on age, sex or general health status, supported by descriptive statistics demonstrating comparability with overall population ⋆**
2. **no selection of cases based on age, sex or general health status, not supported by descriptive statistics ⋆**
3. cases are likely to be biased towards those with more severe COVID-19 symptoms due to selection process – e.g. records from hospitalized patients
4. cases are selected (e.g. based on age or sex) to represent a particular cohort of individuals
5. no description of the derivation of the cohort

<u>Internal validity</u>

Exposure window

B. Ascertainment of exposure

1. **original data collected through interview ⋆**

2. **travel period only ⋆**
3. original data collected through web-based questionnaire with no follow-up of the cases
4. secondary data (using publicly available reports)

C. Precision of the exposure window for cases used in final analysis

1. **includes cases with a range of exposure windows but statistical methods are used to account for this ⋆**
2. includes cases with a range of exposure windows
3. only includes cases with a 1-day exposure window
4. no description/not clear

D. Adjustment for epidemic phase

1. **includes cases collected during a stable epidemic phase ⋆**
2. **includes cases collected during various epidemic phases but proper statistical methods were used for adjustment ⋆**
3. includes cases collected during various epidemic phases but no proper statistical methods were used for adjustment
4. no method/not clear

Outcome

E. Assessment of outcome (onset of symptoms)

1. **original data collected through interview ⋆**
2. original data collected through web-based questionnaire
3. no description/not clear

F. Precision of estimate of outcome

1. **precise date ⋆**
2. time window
3. no description/not clear

Second, we calculate the score of each included study respective to each Omicron subvariant.

Omicron BA.1 variant

Quality assessment item score (A-F)

Study	Quality assessment item score						
	A	B	C	D	E	F	Σ ⋆
Helmsdal *et al*. 2022 [35]	4	1	3	4	1	1	3
Backer *et al.* 2022 [12]	2	4	2	3	1	1	3
Tanaka *et al*. 2022 [18]	1	1	3	1	1	1	5
Del Águila-Mejía *et al*. 2022 [36]	3	1	4	1	1	1	4
Manica *et al*. 2022 [13]	1	1	3	1	1	1	5
Mefsin *et al.* 2022 [37]	2	1	3	2	1	1	5
Liu Y *et al.* 2022 [38]	2	4	1	1	1	1	5
Xin *et al.* 2023 [15]	2	1	1	2	1	1	6
Ward *et al.* 2023 [39]	2	1	3	2	1	1	5
Ogata *et al.* 2023 [19]	1	1	3	4	1	1	4
Guo *et al.* 2023 [40]	1	1	1	2	1	1	6
Zeng *et al.* 2023 [22]	1	1	3	2	1	1	5
Galmiche *et al.* 2023 [14]	3	3	2	3	2	2	0
Park *et al.* 2023 [17]	2	4	1	2	1	1	5
Liu *et al.* 2023 [41]	1	1	1	1	1	1	6
Russell *et al.* 2024 [42]	4	1	1	3	1	1	4
Li *et al.* 2024 [43]	1	1	1	3	1	1	5

Present study	1	1	1	1	1	1	6

<u>Omicron BA.2 variant</u>

Quality assessment item score (A-F)

Study	Quality assessment item score						
	A	B	C	D	E	F	Σ⋆
Mefsin *et al.* 2022 [37]	2	1	3	2	1	1	5
Ward *et al.* 2023 [39]	2	1	3	2	1	1	5
Liu Y *et al.* 2022 [38]	2	4	1	1	1	1	5
Wei *et al.* 2023 [44]	4	1	1	4	1	1	4
Guo *et al.* 2023 [40]	1	1	1	2	1	1	6
Liu *et al.* 2023 [41]	1	1	1	1	1	1	6
Russell *et al.* 2024 [42]	4	1	1	3	1	1	4
Li *et al.* 2024 [43]	1	1	1	3	1	1	5

<u>Omicron BA.5 variant</u>

Quality assessment item score (A-F)

Study	Quality assessment item score						
	A	B	C	D	E	F	Σ⋆
Xiong *et al.* 2023 [45]	1	1	1	1	1	1	6
Ogata *et al.* 2023 [19]	1	1	3	4	1	1	4
Wang *et al.* 2023 [46]	1	1	1	2	1	1	6
Overton *et al.* 2024 [47]	4	3	1	1	2	1	3

E. RANDOM EFFECT MODEL FOR META-ANALYSIS

For each selected study i of a given Omicron variant j, we extracted the estimated mean incubation period $m_{i,j}$ and its 95% confidence or credible intervals (CrI), $(l_{i,j}, u_{i,j})$, or SD, $SD_{i,j}$, and cohort size $N_{i,j}$. The standard error of the mean $\sigma_{i,j}$ was calculated from 95% CrIs according to the following approximation [16]:

$$\sigma_{i,j} = \begin{cases} \dfrac{u_{i,j} - l_{i,j}}{2 \times t_{0.975,N-1} \times \sqrt{N_{i,j}}} & \text{if 95\% CrI is given} \\ \dfrac{SD_{i,j}}{\sqrt{N_{i,j}}} & \text{otherwise} \end{cases}$$

where $t_{0.975,N-1}$ is the 97.5th percentile of Student-t distribution with $(N-1)$ degrees of freedom. When $N \gg 1$, the Student-t distribution approaches the normal distribution and $t_{0.975,\infty} = 1.96$. According to the random-effect model of meta-analysis, each observed mean follows a normal distribution

$$m_{i,j} \sim \text{Normal}(\text{mean} = \theta_{i,j}, \text{SD} = \sigma_{i,j})$$

centered around the "true" mean $\theta_{i,j}$ with standard deviation (SD) equal to the observed standard error $\sigma_{i,j}$. However, the true means approximate some underlying population mean m_j specific to each variant and defined by the formula

$$\theta_{i,j} \sim \text{Normal}(\text{mean} = m_j, \text{SD} = \tau_j)$$

where the parameter τ_j identifies the between-study heterogeneity. The last two formulas can be combined into one by writing

$$m_{i,j} \sim \text{Normal}\left(\text{mean} = m_j, \text{SD} = \sqrt{\sigma_{i,j}^2 + \tau_j^2}\right)$$

For estimating the overall mean across all variants, μ, an additional step is performed by suggesting that m_j also centered around the overall population mean m:

$$m_j \sim \text{Normal}(\text{mean} = m, \text{SD} = \tau)$$

where the parameter τ describes the between-variant heterogeneity.

For the Bayesian inference of the model parameters, we imposed weakly informative priors

$$m_j, m \sim \text{Normal}(\text{mean} = 4, \text{SD} = 8)$$

$$\tau_j, \tau \sim \text{Cauchy}(\text{location} = 0, \text{scale} = 5)$$

SUPPLEMENTARY REFERENCES

56. Reich NG, Lessler J, Cummings DAT, Brookmeyer R. Estimating incubation period distributions with coarse data. *Statistics in Medicine*. 2009;28(22):2769-84. http://doi.org/10.1002/sim.3659

F. SUPPLEMENTARY TABLES AND FIGURES

Supplementary Table 1. Posterior predictive incubation period and estimated model parameters for each distribution (first column). The values are the posterior means, while the 95% credible intervals are shown in the parenthesis. GGD stands for the generalized gamma distribution.

Distribution	Posterior predictive, days	Location	Shape	Scale
gamma	3.48 (1.47-6.26)	-	9.32 (4.54-16.80)	0.42 (0.21-0.76)
Weibull	3.48 (1.05-6.20)	-	2.99 (2.12-4.08)	3.90 (3.39-4.42)
lognormal	3.48 (1.60-6.52)	1.18 (1.03-1.32)	-	0.34 (0.24-0.48)
GGD	3.48 (0.97-6.30)	1.37 (1.20-1.53)	1.05 (0.55-1.60)	0.35 (0.25-0.50)

Supplementary Table 2: Estimated parameters of the incubation period using mixture model, stratified by vaccination status or by age group. The values are shown by their posterior medians and 95% credible intervals indicated in the parenthesis.

	Mean, days	Standard deviation, days	Median, days
**Vaccination**			
Yes	3.25 (2.67-3.92)	1.18 (0.77-2.09)	3.08 (2.46-3.69)
No	4.89 (3.30-20.24)	2.76 (1.02-34.11)	4.35 (2.82-10.99)
**Age group**			
0-17	4.93 (2.22-28.57)	4.10 (0.96-64.58)	3.83 (1.35-14.64)
18-49	3.29 (2.85-3.76)	0.93 (0.62-1.48)	3.19 (2.72-3.65)
50+	4.42 (2.74-11.62)	2.68 (1.12-14.64)	3.74 (2.01-6.40)

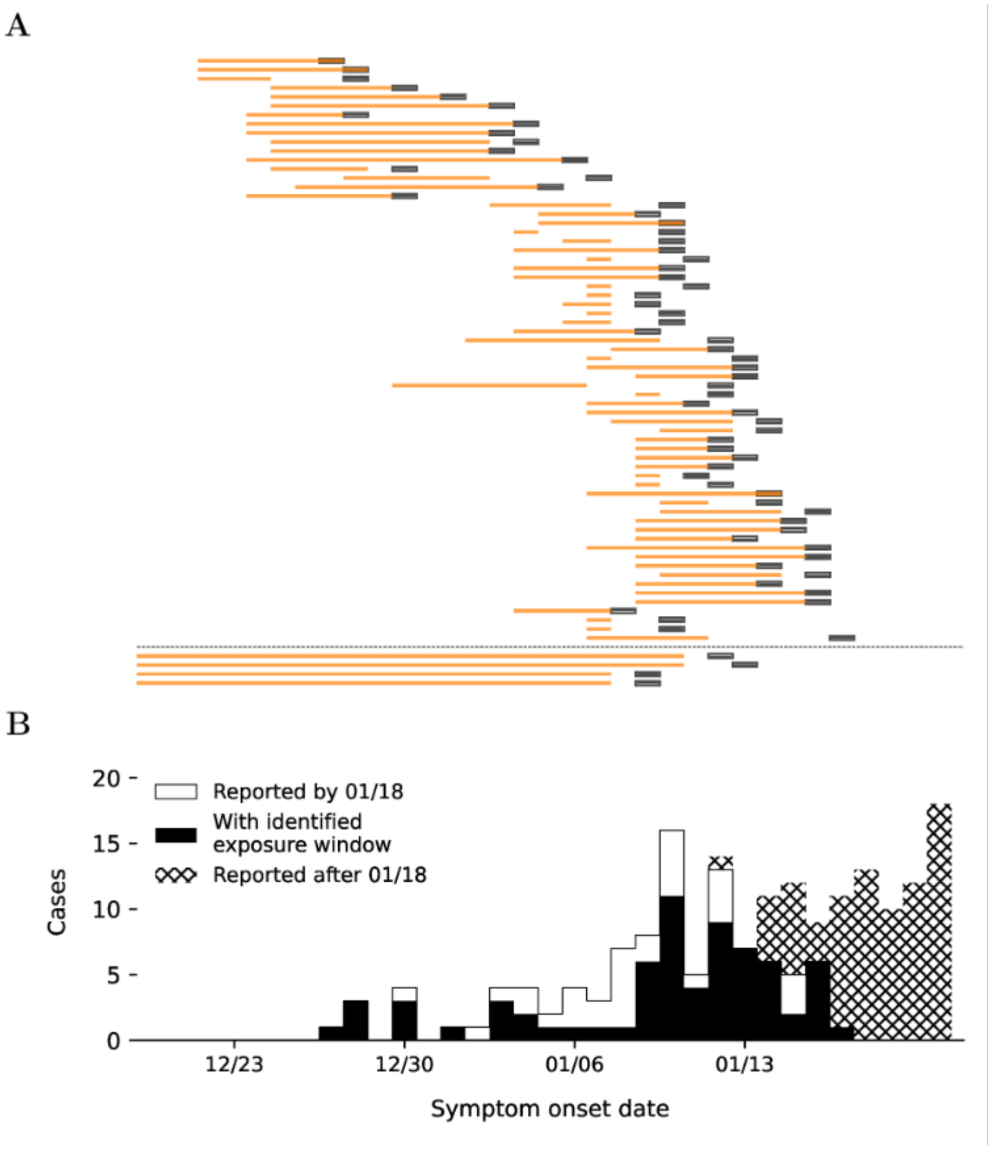

Supplementary Figure 1. Data on confirmed local COVID-19 cases associated with Omicron BA.1 variant that was collected in Taiwan from 25 December 2021 through 18 January 2022. (**A**) shows the exposure windows (in orange) and symptom onset days (in black) for 69 cases included in our study. The 4 cases below the dashed line have left-censored exposure windows (below). (**B**) shows the epidemiological curve for symptomatic COVID-19 cases by their date of symptom onset. Black bars indicate included cases, white bars show cases excluded for insufficient data, and hatched bars cases reported after the cut-off date of January 18, 2022. The horizontal axis is shared among both panels (A) and (B). Cases associated with the COVID-19 cluster among migrant workers were omitted in (B) as non-community related.

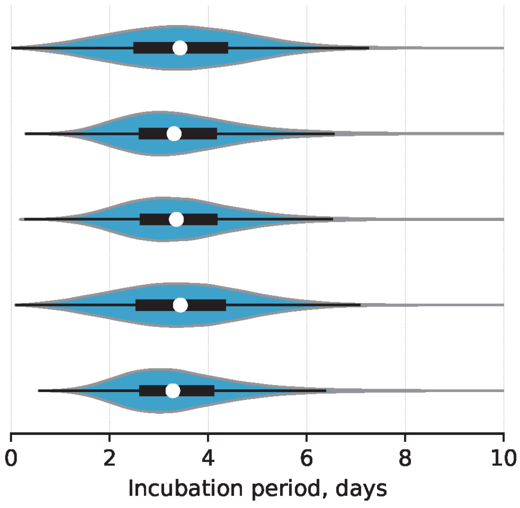

	Mean, days	SD, days	Posterior predict., days	Relative weight, %
GGD	3.47 (2.99-3.97)	1.35 (1.02-1.80)	3.48 (0.96–6.30)	–
mixture	3.49 (3.04-3.98)	1.26 (0.88-1.84)	3.49 (1.51–6.55)	–
gamma	3.48 (3.03-3.94)	1.19 (0.85-1.64)	3.48 (1.47–6.26)	30.4
Weibull	3.48 (3.01-3.96)	1.29 (1.00-1.71)	3.48 (1.05–6.20)	4.6
lognormal	3.48 (3.02-3.95)	1.23 (0.85-1.83)	3.48 (1.60–6.52)	65.0

Supplementary Figure 2. Comparing the estimates of incubation period across various models. The incubation period was fit to the generalized gamma distribution (GGD), standalone gamma, Weibull, or lognormal distribution, or the mixture of last three. The last column shows relative weights for each standalone distribution determined by the inference of the mixture model. Their sum was normalized to one. Inside the violin plots, the circles show the estimated posterior median, thick lines show the interquartile ranges and thin whiskers show the 95% credible intervals. Each value in the table indicates the posterior mean and 95% credible interval that are shown in parenthesis.

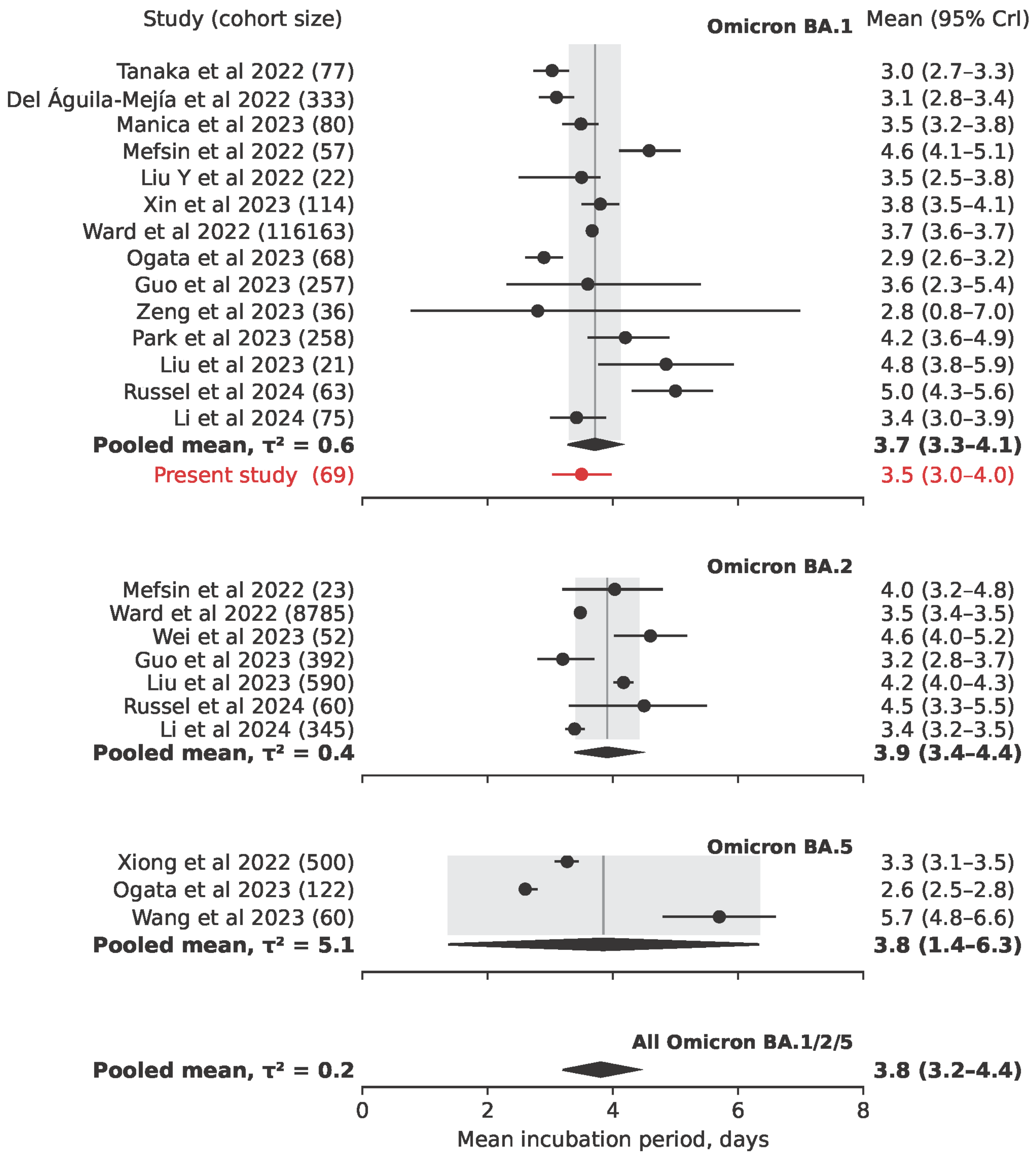

Supplementary Figure 3. Meta-analysis of the mean incubation periods for Omicron variants BA.1, BA.2, and BA.5 when only studies quality assessment scores above four were selected. The pooled mean is indicated in bold, while the estimate of the present study is indicated in red, and it was not a part of the meta-analysis.
